# Hospitalizations and emergency department visits trends among elderly individuals in proximity to death: a retrospective population-based study

**DOI:** 10.1038/s41598-021-00648-1

**Published:** 2021-11-02

**Authors:** Claudio Barbiellini Amidei, Silvia Macciò, Anna Cantarutti, Francesca Gessoni, Andrea Bardin, Loris Zanier, Cristina Canova, Lorenzo Simonato

**Affiliations:** 1 Department of Cardio-Thoraco-Vascular Sciences and Public Health, University of Padua, 35131 Padua, Italy; 2 Division of Biostatistics, Epidemiology and Public Health, Department of Statistics and Quantitative Methods, University of Milano-Bicocca, Milan, Italy; 3 National Centre for Healthcare Research and Pharmacoepidemiology, University of Milano-Bicocca, Milan, Italy; 4 Epidemiological Service, Health Directorate, Friuli-Venezia Giulia Region, Udine, Italy

**Keywords:** Geriatrics, Health policy, Health services, Public health, Quality of life

## Abstract

Acute healthcare services are extremely important, particularly during the COVID-19 pandemic, as healthcare demand has rapidly intensified, and resources have become insufficient. Studies on specific prepandemic hospitalization and emergency department visit (EDV) trends in proximity to death are limited. We examined time-trend specificities based on sex, age, and cause of death in the last 2 years of life. Datasets containing all hospitalizations and EDVs of elderly residents in Friuli-Venezia Giulia, Italy (N = 411,812), who died between 2002 and 2014 at ≥ 65 years, have been collected. We performed subgroup change-point analysis of monthly trends in the 2 years preceding death according to sex, age at death (65–74, 75–84, 85–94, and ≥ 95 years), and main cause of death (cancer, cardiovascular, or respiratory disease). The proportion of decedents (N = 142,834) accessing acute healthcare services increased exponentially in proximity to death (hospitalizations = 4.7, EDVs = 3.9 months before death). This was inversely related to age, with changes among the youngest and eldest decedents at 6.6 and 3.5 months for hospitalizations and at 4.6 and 3.3 months for EDVs, respectively. Healthcare use among cancer patients intensified earlier in life (hospitalizations = 6.8, EDVs = 5.8 months before death). Decedents from respiratory diseases were most likely to access hospital-based services during the last month of life. No sex-based differences were found. The greater use of acute healthcare services among younger decedents and cancer patients suggests that policies potentiating primary care support targeting these at-risk groups may reduce pressure on hospital-based services.

## Introduction

The recent COVID-19 pandemic has strained public healthcare systems worldwide, by rapidly increasing the demand for acute healthcare support. This situation has led to a saturation of healthcare services and a lack in availability of vital support for individuals who cannot autonomously breathe because of acute respiratory distress syndrome. The increasing spread of COVID-19 through the population will require potentiating public health measures such as social distancing and vast use of personal protective equipment or even stronger measures such as new lockdowns. The uncontrolled spread of the pandemic might lead to harsh and ethically challenging situations such as deciding who will or will not receive vital support.

Even in prepandemic times, the demographic shift of aging populations alone in Western countries was expected to increase the demand for healthcare services^1^. Hospital care and emergency department visits (EDVs) are fundamental services of any healthcare system. At the same time, hospital admissions and EDVs impact public health in terms of both individual quality of life and healthcare costs^2–4^, and their utilization intensifies significantly at the end of life^1,5–7^. Previous literature indicates that approximately 84% of people who die were hospitalized the year before death, with variations depending on age and sex^1,8^, and 70% accessed an emergency department in their last 12 months of life^7^. A progressive decline in general health conditions commonly precedes death, particularly for individuals dying from chronic diseases. Several factors influencing the use of healthcare services toward the end of life have been investigated, particularly focusing on hospital admissions. Younger age^9^, respiratory diseases and respiratory symptoms^10^, and the absence of adequate support by palliative care networks^11,12^ have been found to be positively associated with a heavier use of healthcare services at the end of life. However, trends of acute healthcare service utilization in proximity to death and the factors influencing these trends, have not been thoroughly examined at the population level.

This study aimed to analyze monthly acute healthcare service utilization during the last two years of life, to examine possible differences in trends based on sex, age and main cause of death, and to identify, in prepandemic times, precise time frames when these changes occurred at a population level.

## Methods

### Study population

We collected the electronic health records of all elderly residents (411,812 individuals) between January 1st 2000 and December 31st 2014, living in the Friuli-Venezia Giulia region of northeastern Italy. Data with demographic information on all residents insured by the National Health Service (NHS) are held in the population health register. Using death certificates, we identified all individuals aged 65 years of above who died between 2000 and 2014 (162,125 decedents). Based on the population registry, all the residents who were not continuously registered in the region during the 2 years prior to death (19,291 individuals) were excluded from the analyses, leading to a final population of 142,834 decedents. The main characteristics considered in our analyses among individuals included and excluded from the study were similarly distributed (data not shown). The decedents were then linked via anonymous identification codes to hospitalization and EDV electronic health records, which were automatically recorded at a regional data collection center. Given the universalistic healthcare model of the Italian NHS, information from these databases fully reflects the actual use of healthcare resources from the entire population. No informed consent or ethics committee approval was required because record linkage in this study was based on computerized databases of medical records with all the data being anonymized before analyses, according to the Italian guidelines for the conduction of observational studies on pharmacological exposures^13^.

### Outcome

Hospitalization records were used to determine admission and discharge dates and then to calculate the duration of hospitalization episodes. Day-hospital services were excluded from the analyses. All other hospitalizations (unplanned and planned) were included. An individual was considered hospitalized in a 30-day period (from here on “month”) after having spent at least one day in a hospital department during that period (i.e., if a hospitalization overlapped two months, the individual was considered hospitalized in both months). The proportion of hospitalized subjects was then calculated for each month. The frequency, mean, standard deviation (SD), median, and interquartile range (IQR) of the number of hospitalizations were calculated for each month, while the hospitalization length was calculated at 12 months, 6 months and 1 month before death.

The electronic health records of all EDVs were used to determine the dates in which any individual accessed the emergency department. The proportion of individuals with ≥ 1 EDV was then calculated for all 24 months before death.

Subgroup analyses were performed based on sex, age at death (categorized as 65–74, 75–84, 85–94, and ≥ 95 years), and main cause of death (coded according to the International Classification of Diseases—9th Revision—Clinical Modification (ICD-9-CM)). We considered only the three most frequent main causes of death in our population: cardiovascular diseases (ICD-9-CM: 390–459), cancer (ICD-9-CM: 140–239) and respiratory diseases (ICD-9-CM: 460–519). We further analyzed the three most frequent specific causes of death within these broader categories. We also calculated the overall proportion of hospitalized subjects and those who accessed the emergency department during the last 24 months, 12 months, 6 months, and 1 month of life.

### Statistical analyses

Nonlinear regression models were fitted to the monthly proportion of hospitalized patients and EDVs, to estimate change points. Change point analysis is based on two segments that connect in a smooth fashion and considers the mean of Y as a quadratic function in X, for values of X less than X_0_, and the mean of Y as a constant for values of X greater than X_0_. Therefore, the constant is the mean function (plateau). Conditions imposed on the model for the two segments are as follows: the curve must be continuous, and the curve must be smooth. These segmented models can be fit even when the change point (X_0_) is not known. Consequently, the change point can be estimated as the intersection between the quadratic and the plateau segment. The parameters were estimated by nonlinear least squares. Wald-based formula was used to calculate 95% confidence intervals. For each model we report the change-point and its approximate 95% confidence interval.

P-value for differences in the number of individuals with at least one hospitalization or at least one EDV during the 24 months, 12 months, 6 months and 1 month preceding death were estimated using a chi-square test.

### Sensitivity analyses

Triage color tags are attributed by a specifically trained nurse upon arrival at the emergency department. These color tags are used to stratify assistance to patients based on the presence of life-threatening and urgent conditions, or nonurgent conditions that do not require immediate medical support. In a subanalysis focusing on EDVs, we included only individuals with triage color tags corresponding to urgency (yellow), emergency (red) or deceased (black) (excluding nonurgent (white) and minor urgency (green)). By doing so, we wanted to examine only severe health conditions that are unlikely to be modified by primary and long-term care support (that were not available for our analyses).

To quantify a possible effect of time, we also considered differences by calendar year (2002–2005, 2006–2009, and 2010–2014).

All analyses were performed using Statistical Analysis System software (version 9.4; SAS Institute, Cary, North Carolina) and the SAS NLIN procedure for change point analyses. The statistical significance was set at the 0.05 level. All *p* values were 2‐sided.

## Results

Among all elderly residents in Friuli-Venezia Giulia (N = 411,812), we identified 142,834 individuals aged 65 years or more, that died between 2002 and 2014 (Table 1). The mean age of the decedents was 83.6 years (SD 8.4), with 80,137 (56.1%) women, and the two most represented age groups were 75–84 years (35.7%) and 85–94 years (38.3%). The most frequently observed main cause of death was cardiovascular diseases (39.7%), followed by cancer (28.2%) and respiratory diseases (9.9%). Overall, 353,481 hospitalizations and 380,155 EDVs were recorded during the 2 years preceding death.

**Table 1:** Study population size and proportion of individuals who were hospitalized and had accessed the emergency department 24, 12, 6, and 1 month before death.

	Study population N (%)	Hospitalizations %				Emergency department visits %			
		24 months	12 months	6 months	1 month	24 months	12 months	6 months	1 month
**Total**	142,834 (100.0)	86.5	82.4	78.6	63.7	85.8	81.2	76.6	55.8
**Sex**									
Female	80,137 (56.1)	84.3	79.8	75.8	60.5	84.7	79.8	74.9	54.2
Male	62,697 (43.9)	89.3	85.6	82.1	67.8	87.2	83.1	78.7	57.8
*P* value	< .0001	< .0001	< .0001	< .0001	< .0001	<.0001	< .0001	< .0001	<.0001
**Age**									
65–74	24,899 (17.4)	89.5	86.3	83.0	69.0	84.3	80.2	75.6	52.7
75–84	50,964 (35.7)	89.9	86.3	82.8	68.1	87.7	83.6	79.3	57.5
85–94	54,739 (38.3)	85.3	80.7	76.5	60.9	86.6	81.8	77.0	57.3
95 +	12,232(8.6)	71.9	65.4	60.9	47.2	78.0	70.9	65.3	48.1
*P* value	< .0001	< .0001	< .0001	< .0001	< .0001	< .0001	< .0001	< .0001	< .0001
**Cause of death**									
Cancer	40,343 (28.2)	95.7	93.2	89.8	72.4	88.7	85.3	80.9	53.7
Cardiovascular	56,687 (39.7)	80.1	74.5	69.8	54.6	82.5	76.8	71.4	52.1
Respiratory	14,108 (9.9)	92.3	89.5	87.3	77.2	91.6	88.5	85.7	72.6
Other	31,696 (22.2)	83.6	79.5	75.9	66.9	85.5	80.9	76.3	57.5
*P* value	< .0001	< .0001	< .0001	< .0001	< .0001	< .0001	< .0001	< .0001	< .0001

### Hospitalizations

**Figure 1:**
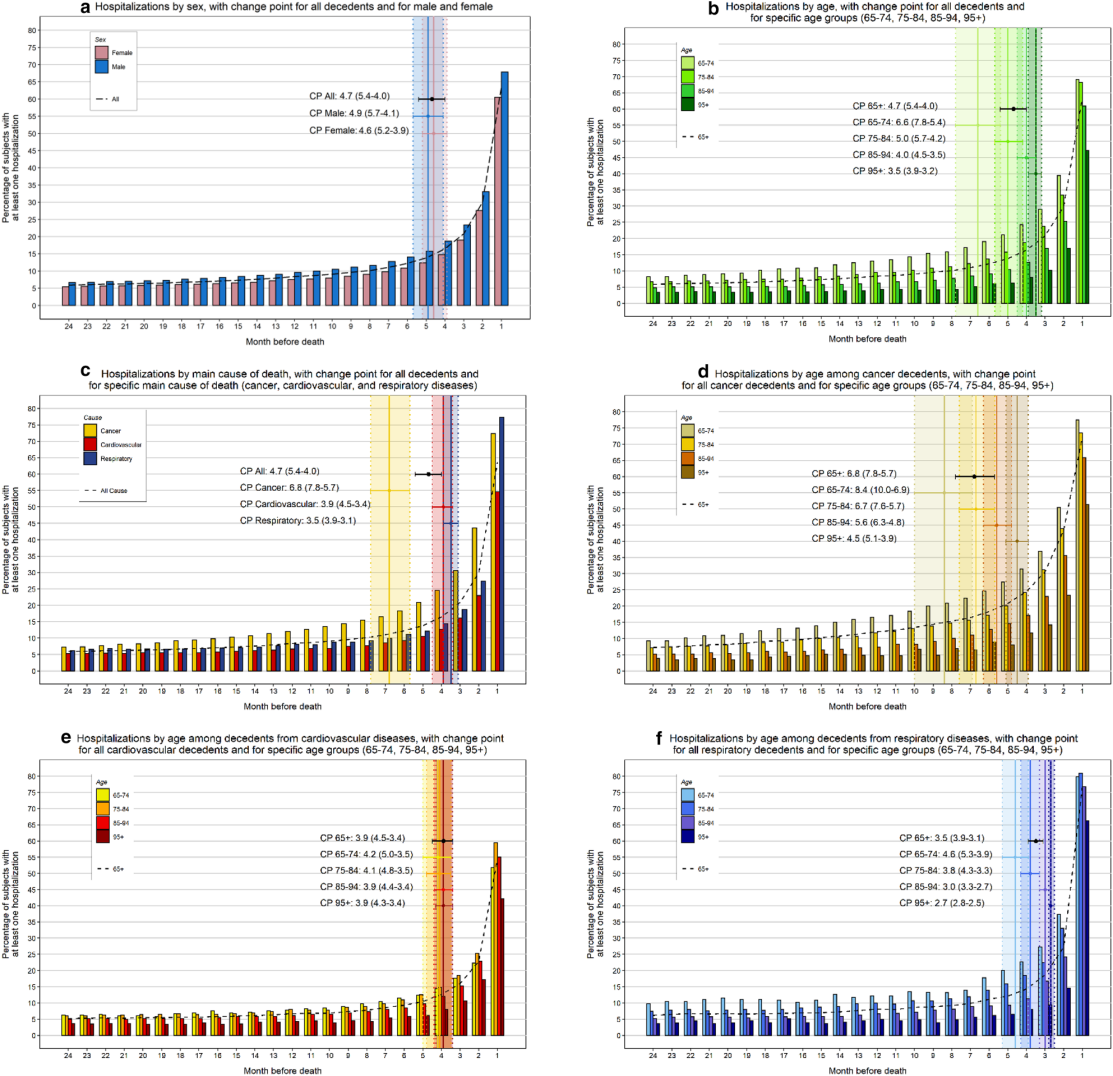
Proportion of hospitalized decedents stratified by sex, age and main cause of death, in the 24 months preceding death, with specific change-point month and 95% confidence interval.

In the month before death, the proportion of individuals hospitalized at least once was 63.7%, while this cumulative proportion did not vary substantially from 12 (82.4%) to 24 months (86.5%) before death (Table 1). Overall, men were more likely to be hospitalized than women (89.3% and 84.3% respectively). In the 2 years preceding death, the proportion of hospitalized individuals was relatively similar among decedents aged 65 to 94 years(ranging from 85.3 to 89.9%), but it was significantly lower in the oldest group, aged 95 years or older (71.9%). In the 24 months preceding death, cancer decedents were most likely to be hospitalized at least once (95.7%), followed by decedents from respiratory diseases (92.3%), and cardiovascular diseases (80.1%). Approximately more than half individuals (54.3%) had died in a hospital facility (data not shown). The proportion of hospitalized subjects remained relatively low (≤ 15%) and stable during most of the two-year period analyzed (Fig. 1a), with an exponential rise starting at 4.7 months before death (Table 2).

**Table 2:** Change-point analysis indicating the month before death when the proportion of individuals who were hospitalized and had accessed the emergency department exponentially increased.

	Hospitalizations change-point month (95% CI)		Emergency department visits change-point month (95% CI)	
**Sex**				
Female	4.6	(3.9–5.2)	3.8	(3.3–4.3)
Male	4.9	(4.1–5.7)	4.0	(3.4–4.5)
All	4.7	(4.0–5.4)	3.9	(3.3–4.4)
**Age**				
65–74	6.6	(5.4–7.8)	4.6	(3.9–5.3)
75–84	5.0	(4.2–5.7)	4.0	(3.4–4.6)
85–94	4.0	(3.5–4.5)	3.7	(3.2–4.1)
≥ 95	3.5	(3.2–3.9)	3.4	(3.0–3.7)
**Cause of death**				
**Cancer**				
All age groups	6.8	(5.7–7.8)	5.8	(5.0–6.6)
65–74	8.4	(6.9–10.0)	5.9	(5.1–6.7)
75–84	6.7	(5.7–7.6)	5.9	(5.1–6.8)
85–94	5.6	(4.8–6.3)	5.6	(4.9–6.3)
≥ 95	4.5	(3.9–5.1)	4.7	(4.1–5.4)
**Cardiovascular**				
All age groups	3.9	(3.4–4.5)	3.5	(3.0–3.9)
65–74	4.2	(3.5–5.0)	2.8	(2.4–3.2)
75–84	4.1	(3.5–4.8)	3.5	(3.0–4.0)
85–94	3.9	(3.4–4.4)	3.6	(3.1–4.0)
≥ 95	3.9	(3.4–4.3)	3.7	(3.3–4.1)
**Respiratory**				
All age groups	3.5	(3.1–3.9)	2.9	(2.6–3.2)
65–74	4.6	(3.9–5.3)	3.6	(3.1–4.1)
75–84	3.8	(3.3–4.3)	3.3	(2.9–3.7)
85–94	3.0	(2.7–3.3)	2.8	(2.6–3.1)
≥ 95	2.7	(2.5–2.8)	2.7	(2.5–2.8)

No relevant differences were found between male individuals (change point: 4.9 months prior to death) and female individuals (change point: 4.6 months before death) regarding the comparison of their trends (Fig. 1a). Regardless of the cause of death, hospitalizations were inversely related to age (Fig. 1b). Changes in trends began further away from death among subjects aged 65–74 (change point 6.6 months) than among those aged ≥ 95 years (change point 3.5 months) (Table 2).

Analysis of disease-specific mortality revealed that the proportion of hospitalized individuals was strongly affected by the main cause of death (Fig. 1c). Trends among cancer decedents showed a consistently higher proportion of hospitalized subjects, while decedents from cardiovascular and respiratory diseases had similar trends. The exponential rise in the trend began closer to death for decedents from cardiovascular (change point: 3.9 months) and respiratory diseases (change point: 3.5 months), than in cancer decedents (change point: 6.8 months) (Table 2). When analyzing the change in hospitalizations from the second to the last month of life, we observed a more than twofold increase in the proportion of hospitalized individuals among decedents from cardiovascular (22.9–54.6%) and respiratory diseases (27.3–77.2%), while the increase was less marked among cancer decedents (43.5–72.4%). Decedents from respiratory diseases were most likely to be hospitalized in the last month of life (77.2%). Hospital use among decedents from cancer and respiratory diseases, showed a strong inverse relationship with age throughout the entire period, although this was not as evident for cardiovascular diseases (Fig. 1d–f). The analysis for specific cause of death confirmed cardiovascular and respiratory diseases were more common acute conditions (Supplemental Table 1). As death approached, the number of hospitalizations increased, as did the mean length of hospitalizations (Supplemental Tables 2 and 3). We observed no differences during the study period in trends by calendar year (Supplemental Figure 1).

### Emergency department visits

Similar to hospitalizations, the proportion of subjects who had accessed the emergency department at least once increased progressively during the 24 months that preceded death. A total of 55.8% had an EDV the month before death, while no significant differences were found between individuals with at least one EDV during the 24 (85.8%) and 12 months (81.2%) preceding death (Table 1). Men were more likely to access the emergency department (87.2%) than women (84.7%), and younger age groups (65–94 years) were more likely to have at least one EDV (84.3–87.7%) than those aged ≥ 95 years (78.0%). Decedents from respiratory diseases were most likely to have an EDV (91.6%), followed by decedents from cancer (88.7%) and cardiovascular diseases (82.5%). The change in the EDV trend in the overall population began 3.9 months before death (Table 2).

**Figure 2:**
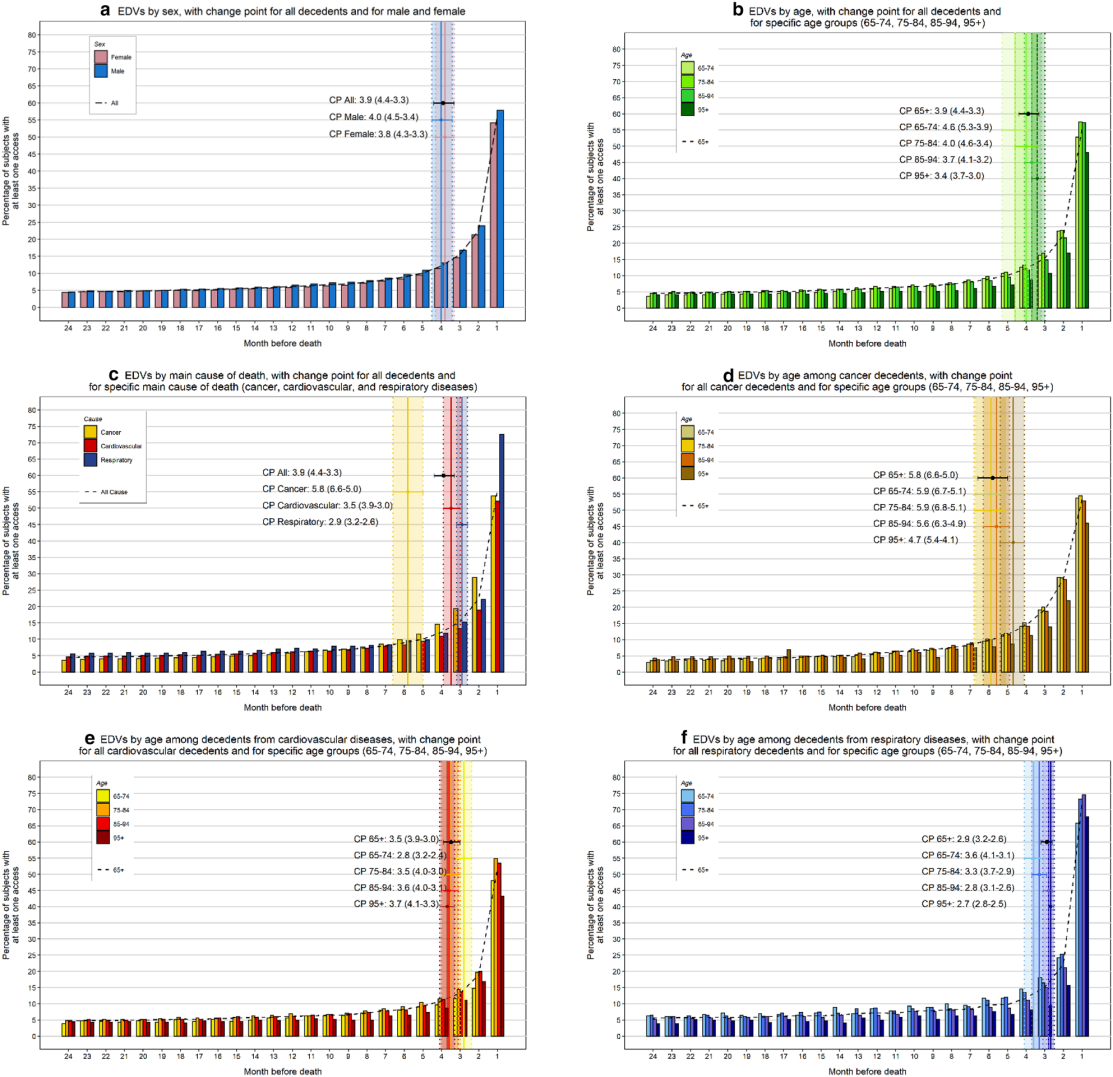
Proportion of decedents with an emergency department visit (EDV), stratified by sex, age and main cause of death in the 24 months preceding death, with specific change-point month and 95% confidence interval.

No relevant sex differences were present (Fig. 2a) as confirmed by similar change points in men (4.0 months) and women (3.8 months). Among the eldest group (≥ 95 years), a markedly lower proportion of individuals accessed the emergency department (Fig. 2b), with a closer change-point to death (3.4 months) (Table 2).

Decedents from cardiovascular and respiratory diseases had relatively similar EDV trends (Fig. 2c) and change points (3.5 and 2.9 months, respectively), while the exponential increase occurred much earlier among cancer decedents (5.8 months) (Table 2). Similar to hospitalizations, decedents from respiratory diseases were most likely to access the emergency department the month before death (approximately 72.6%). After performing a subgroup analysis by main cause of death, we found no clear age-related trend, apart from a significantly lower proportion of subjects accessing the emergency department in the oldest age group (≥ 95 years) (Fig. 2d–f). The number of EDVs per decedent also increased in proximity to death (Supplemental Table 3). No significant differences by calendar year have been observed (Supplemental Figure 1). Finally, after excluding nonurgent or minor urgencies, we found no significant differences in the EDV trends observed previously (Supplemental Figure 2).

## Discussion

In the present study, we observed an exponential increase in the number of individuals who required hospitalization (starting 4.7 months before death) or accessed the emergency department (starting 3.9 months before death). This increase was mostly evident, and occurred farther away from death, particularly among younger decedents and cancer patients. Previous studies have also observed a similar exponential change in acute healthcare service use when approaching death. However, to the best of our knowledge, no previous study has applied a change-point analysis to identify exponential changes in these trends across specific subgroups.

Hospitalizations and EDVs are highly impacting events, both regarding individual quality of life and resource consumption for the NHS^2–4^. A detailed analysis of acute healthcare utilization trends in nonpandemic times is useful to better understand phenomena that occur at the end of life, on a population level, and identify the main drivers for different patterns of acute healthcare utilization. Although the time of an individual’s death cannot be predicted, estimates on the number of expected deaths in a population are frequently provided. A well-defined epidemiological overview of the prevalence and incidence of noncommunicable diseases, combined with disease-specific mortality forecasts in a population, may also provide useful estimates of the need for healthcare services in the near future. This retrospective approach serves to describe patterns of healthcare service use in proximity to death and provided insights for healthcare policy-makers on the expected need for hospital-based healthcare services in a specific population. Previous literature has suggested that closeness to death may be the main driver of change in the intensity of acute healthcare service utilization^1,5,14^. The period considered for the end of life varies in the literature, ranging from days to years before death^7,15–18^ and mainly depending on the specific conditions considered (i.e., studies on cancer patients generally require a longer time frame)^19–22^. At the population level, appropriate overall and disease-specific time frames can be defined to retrospectively analyze phenomena that occur at the end of life based on changes in acute healthcare utilization trends, defined by specific change points. This information allows a more accurate guidance on retrospective analyses to assess the impact of public healthcare policies on the utilization of hospital-based services. Previous literature has observed that sex, age and cause of death are responsible to a variable extent for different healthcare utilization trends at the end of life^1,23^. In the present study, we observed an overall higher use among men, but highly comparable trends among men and women, as confirmed by similar change points. Several studies have shown that older age is associated with lower use of acute healthcare services at the end of life^1,5,6,23,24^. This finding can be linked to a high proportion of institutionalized individuals who are regularly provided with basic assistance, potentially reducing the need to access hospital-based services, if not in close proximity to death, as suggested by the change point. Another explanation could lie in the less aggressive medical care delivered to older individuals^25^. Additionally, individuals who died at a very old age might have survived and reached said age because of fewer health problems, which could be attributed to a more favorable genetic and environmental background^26^. Frailty can also play a role in increasing vulnerability to the onset of acute conditions, rapidly leading to death, concentrating the most acute healthcare support in close proximity to death^27–29^. People at the end of life also frequently show symptoms that lead them to seek medical support^30^. A better understanding of these different factors can indirectly provide hints for future research to help policy makers deliver appropriate interventions to high-risk subgroups, for example promoting primary care support to reduce the use of acute healthcare services for nonurgent conditions.

### Hospitalizations

The overall number of hospitalized individuals was stable up to 6 months before death, when an exponential increase was found in proximity to death, as observed in previous literature^1,23^. The high number of hospitalized individuals in the last month of life is also related to deaths in the hospital (45.8%), as observed in other contexts^31,32^. Consistent with previous literature, we observed no significant differences in the trends among men and women^23^; therefore, we did not apply sex stratification to any analysis. Age appeared to be a major driver of differences in hospitalization trends, with a strong inverse relationship across the entire period. The earlier exponential rise in younger hospitalized individuals is likely related to a higher prevalence of cancer (and its chronic management) toward mid-life^33^. Another hypothesis is that younger individuals may have a greater resistance to health stress, thereby extending the period of health instability, with a consequently longer use of hospital-based services^26^. Cancer decedents, compared with those who die from cardiovascular or respiratory diseases, were most likely to be hospitalized and had the earliest shift to an exponential trend. These findings can be partly explained by a higher prevalence of acute conditions among the broad categories of cardiovascular and respiratory diseases (as suggested by Supplemental Table 1), but the inverse relation with age was consistent across all three macrocategories of main cause of death, although it was more evident among decedents for cancer and respiratory diseases. As expected, the exponential increase in the proportion of individuals with at least one hospitalization per month relates to the total number of hospitalizations per individual, which increases in proximity to death.

### Emergency department visits

Similar to that observed for hospitalizations, the number of individuals accessing the emergency department was relatively stable throughout the entire period in all subgroup analyses until an exponential rise occurred in the last 4 months of life. The proportion of decedents who had accessed an emergency department is lower than those observed for hospitalizations, because many hospital admissions are scheduled and therefore, are not preceded by an EDV. We found no relevant sex differences, and age-related trend differences were not as marked as those for hospitalizations, with the only consistent finding being the lower proportion of individuals aged ≥ 95 years who had accessed the emergency department (also in subgroup analyses by main cause of death). This finding agrees with most previous literature^1,5,6,23^, although some contrasting results have been reported^34^. Similar to what was observed with hospitalizations, the total number of EDVs per individual underwent a sharp increase as death approached.

### Strengths and limitations

To the best of our knowledge, no study in the literature has examined monthly acute healthcare utilization trends, with subgroup analyses based on sex, age and main cause of death. Most studies on healthcare utilization at the end of life have focused on shorter periods (1 to 6 months) or longer periods considered in their entirety (1 to 2 years), thereby mitigating the strong time-dependent differences within this timeframe. The relatively elevated time resolution over a 2-year period, and the relatively long stable trend for both hospitalizations and EDVs allowed us to properly identify the precise moment when acute healthcare service demand intensified exponentially. Given the population-based study and presence of healthcare services available to all citizens, we are confident that we have a complete overview of hospitalizations and EDVs. Additionally, to our best knowledge, we are the first to have applied change-point analysis to these trends in proximity to death, thereby allowing a more objective and standardized comparison between specific groups (with a greater stratification than the only previous paper that used a similar analysis^7^).

A possible limitation of our study was the absence of cost analyses, although these have been vastly addressed by previous literature that has shown a direct relation to costs^3,4,16,17,35–37^. The differences in healthcare utilization involve significant differences in costs as well (particularly for cancer patients)^38^, related to specific clinical characteristics and intensity of care. Data concerning frailty and the presence of comorbidities were also not available; therefore, specific subgroup analyses could not be performed. Nevertheless, we believe subgroup analyses by main cause of death provide relatively comparable age-specific trends. Another limitation concerns quantitative and qualitative data on hospitalizations and EDVs (i.e., admission diagnosis, or hospitalization department) that have not been considered. Unfortunately, data on hospice and palliative care services were not available for these analyses. This type of healthcare support could contribute to mitigating the use of hospital-based services. However, our sensitivity analysis on EDVs suggests the robustness of our results, in which, excluding nonurgent conditions, we continued to observe trends comparable to those observed for all EDVs. Accessing the emergency department for nonsevere conditions can be linked to insufficient support of primary care, long-term care or home assistance^17^. By observing similar results in our sensitivity analysis, we believe the possible bias derived from the lack of adjustment for these healthcare services should be limited. However, while consistent evidence suggests that among primary care services, contacts with general practitioners are associated with fewer hospitalizations for ambulatory care-sensitive conditions, findings concerning other health conditions, particularly among people with complex needs, have not shown consistent results.

No relevant changes occurred in the access to hospice, palliative care, long-term care or primary care services between 2015 and the outbreak of COVID-19. COVID-19 has led to strong changes in the use of hospital-based services, but the scenario of healthcare service use at the end of life in the times preceding the COVID-19 pandemic was unlikely to be different from the one we observed. Finally, although data were derived from a single region in Italy, the study population was rather vast. Additionally, by performing subgroup analyses we further ensured the generalizability of our results.

Future studies are required to examine trend differences related to specific causes of death. They may also consider healthcare cost variations in proximity to death, using a change-point analysis, focusing on specific clinically defined subgroups. Finally, comparing hospitalizations and EDVs in proximity to death before and after the COVID-19 pandemic, as well as before and after major public health measures (such as lockdowns), can help estimate some of the indirect consequences of COVID-19^39,40^ on public health.

The exponential increase in the number of individuals who access acute healthcare services as approaching death, particularly among younger decedents and cancer patients, suggests possible targets for interventions. Potentiating primary care support, particularly among categories that are more likely to use acute healthcare services, might reduce their use of hospital-based services. Given the very high proportion of decedents from respiratory diseases who access the emergency department and are hospitalized in the last month of life, it may be advisable to potentiate specific healthcare facilities for these patients, exploiting the adaptability that acute healthcare services have shown to be capable of providing during the COVID-19 pandemic.

## Supplementary Information

Supplementary Information.

## Data Availability

The data we used for our study cannot be shared publicly. The permission and process to access the data has been granted exclusively to the researchers of the University of Padua who worked on this study, by the data owner the Epidemiological Service, Health Directorate, Friuli-Venezia Giulia, according to a research agreement, signed by both parts. The permission for other institutions to access the data was not granted by the data owner. Access to data can be granted by the Epidemiological Service, Health Directorate, Friuli-Venezia Giulia Region upon reasonable request, by contacting the corresponding author.
