# Supplementary material for: Hospitalizations and emergency department visits trends among elderly individuals in proximity to death: a retrospective population-based study
Source: Sci Rep. 2021 Nov 2;11:21472. doi: 10.1038/s41598-021-00648-1 (PMC8563963; doi:10.1038/s41598-021-00648-1)
Supplement: Supplementary file 1 — Supplementary Information. [file 41598_2021_648_MOESM1_ESM.docx]

**Hospitalizations and emergency department visits trends among elderly individuals in proximity to death: a retrospective population-based study.**

**Claudio Barbiellini Amidei^1*^, Silvia Macciò^1^, Anna Cantarutti^2^, Francesca Gessoni^1^, Andrea Bardin^1^, Loris Zanier^3^, Cristina Canova^1*^, Lorenzo Simonato^1^**

^1^ Department of Cardio-Thoraco-Vascular Sciences and Public Health, University of Padua, Padua, Italy.

^2^ Department of Statistics and Quantitative Methods, Division of Biostatistics, Epidemiology and Public Health, University of Milano-Bicocca, Milan, Italy; National Centre for Healthcare Research and Pharmacoepidemiology, University of Milano-Bicocca, Milan, Italy.

^3^ Epidemiological Service, Health Directorate, Friuli-Venezia Giulia Region, Udine, Italy.

**Corresponding authors:**

Claudio Barbiellini Amidei
Department of Cardio-Thoraco-Vascular Sciences and Public Health, University of Padua, Padua, Italy.
35131 Padova, Italy
e-mail: [claudioamidei@gmail.com](mailto:claudioamidei@gmail.com)
Phone: +39 049 827 5752
Fax: +39 049 821 8764

Cristina Canova
Department of Cardio-Thoraco-Vascular Sciences and Public Health, University of Padua, Padua, Italy.
35131 Padova, Italy
e-mail: [cristina.canova@unipd.it](mailto:cristina.canova@unipd.it)
Phone: +39 049 827 5391
Fax: +39 049 827 5407

**Supplemental material**

**Supplemental Table 1.** Study population size and proportion of individuals that were hospitalized and that accessed the emergency department by specific cause of death (the three most common causes among cancer, cardiovascular and respiratory diseases), during the 24, 12, 6, and 1 month before death.

|  | **Study population**  **N (%)** | **Hospitalizations %** | | | | **Emergency department visits %** | | | |
| --- | --- | --- | --- | --- | --- | --- | --- | --- | --- |
|  |  | **24**  **months** | **12**  **months** | **6**  **months** | **1**  **month** | **24**  **months** | **12**  **months** | **6**  **months** | **1**  **month** |
| **Specific cause of death (ICD-9-CM code)** |  |  |  |  |  |  |  |  |  |
| **Cancer** | 40,343 (100.0) |  |  |  |  |  |  |  |  |
| Malignant neoplasm of trachea bronchus and lung (162) | 7,363 (18.3) | 97.0 | 95.1 | 92.2 | 79.1 | 90.6 | 87.6 | 84.0 | 59.6 |
| Malignant neoplasm of colon (153) | 3,630 (9.0) | 95.8 | 92.4 | 87.9 | 72.1 | 88.4 | 84.2 | 79.2 | 51.5 |
| Malignant neoplasm of stomach (151) | 2,772 (6.9) | 96.7 | 94.5 | 91.1 | 74.6 | 86.9 | 83.3 | 79.1 | 50.0 |
| Malignant neoplasm of pancreas (157) | 2,772 (6.9) | 97.7 | 96.2 | 93.1 | 77.4 | 90.0 | 87.1 | 82.9 | 52.5 |
| **Cardiovascular** | 56,687 (100.0) |  |  |  |  |  |  |  |  |
| Other forms of chronic ischemic heart disease (414) | 10,394 (18.3) | 82.6 | 75.8 | 70.5 | 57.0 | 84.7 | 78.1 | 72.1 | 50.3 |
| Acute myocardial infarction (410) | 7,555 (13.3) | 71.0 | 64.3 | 59.2 | 48.8 | 74.8 | 67.8 | 62.2 | 47.1 |
| Acute, but ill-defined, cerebrovascular disease (436) | 5,505 (9.7) | 88.2 | 84.9 | 81.5 | 70.2 | 88.3 | 84.7 | 80.8 | 60.6 |
| **Respiratory** | 14,108 (100.0) |  |  |  |  |  |  |  |  |
| Chronic bronchitis (491) | 3,883 (27.5) | 89.4 | 85.0 | 81.4 | 72.2 | 89.0 | 84.3 | 80.1 | 62.8 |
| Bronchopneumonia, organism unspecified (485) | 3,808 (27.0) | 93.2 | 91.8 | 90.1 | 86.5 | 92.6 | 90.7 | 88.7 | 78.0 |
| Pneumonia, organism unspecified (486) | 2,540 (18.0) | 96.6 | 95.3 | 94.3 | 92.0 | 94.4 | 92.8 | 91.5 | 82.3 |

**Supplemental Table 2.** Hospitalization length during specific time frames before death (12, 6, and 1 month).

| **Cause of death** | **12 months** | | | | **6 months** | | | | **1 month** | | | |
| --- | --- | --- | --- | --- | --- | --- | --- | --- | --- | --- | --- | --- |
|  | **Mean** | **(SD)** | **Median** | **(Q3-Q1)** | **Mean** | **(SD)** | **Median** | **(Q3-Q1)** | **Mean** | **(SD)** | **Median** | **(Q3-Q1)** |
| **All causes** | 27.2 | (40.1) | 15 | (35-3) | 20.0 | (26.4) | 12 | (28-1) | 8.0 | (9.5) | 4 | (14-0) |
| **Cancer** | 41.7 | (53.3) | 26 | (48-12) | 29.1 | (31.6) | 20 | (38-8) | 10.2 | (9.9) | 8 | (17-0) |
| **Cardiovascular** | 19.3 | (29.0) | 10 | (26-0) | 14.7 | (21.6) | 7 | (21-0) | 6.3 | (8.8) | 1 | (10-0) |
| **Respiratory** | 25.2 | (33.0) | 16 | (33-5) | 19.3 | (23.7) | 12 | (27-3) | 9.2 | (9.4) | 6 | (15-1) |

**Supplemental Table 3.** Number of hospitalizations and emergency department visits in the cohort of decedents (N = 142,834), at 24, 12, 6, 3, and 1 month before death.

|  | **24 months** | **12 months** | **6 months** | **3 months** | **1 month** |
| --- | --- | --- | --- | --- | --- |
| **Hospitalizations** |  |  |  |  |  |
| Frequency | 6,381 | 8,745 | 12,692 | 20,753 | 117,172 |
| Cumulative frequency | 353,481 | 255,131 | 198,504 | 156,506 | 117,172 |
| Mean (SD) | 2.86 (2.16) | 2.97 (2.21) | 3.00 (2.22) | 2.97 (2.20) | 2.77 (2.08) |
| Median (Q1-Q3) | 2 (1-3) | 2 (1-3) | 2 (1-3) | 2 (1-3) | 2 (1-3) |
| **Emergency department visits** |  |  |  |  |  |
| Frequency | 7,356 | 10,107 | 14,802 | 26,074 | 95,914 |
| Cumulative frequency | 380,155 | 280,435 | 211,698 | 159,823 | 95,914 |
| Mean (SD) | 3.10 (2.63) | 3.20 (2.65) | 3.21 (2.60) | 3.17 (2.55) | 3.04 (2.45) |
| Median (Q1-Q3) | 2 (1-3) | 2 (1-3) | 2 (1-3) | 2 (1-3) | 2 (1-3) |

**Supplemental Figure 1.** Proportion of individuals hospitalized and accessing the emergency department in the 24 months preceding death, stratified by calendar year (2002-2005, 2006-2009, and 2010-2014).

| 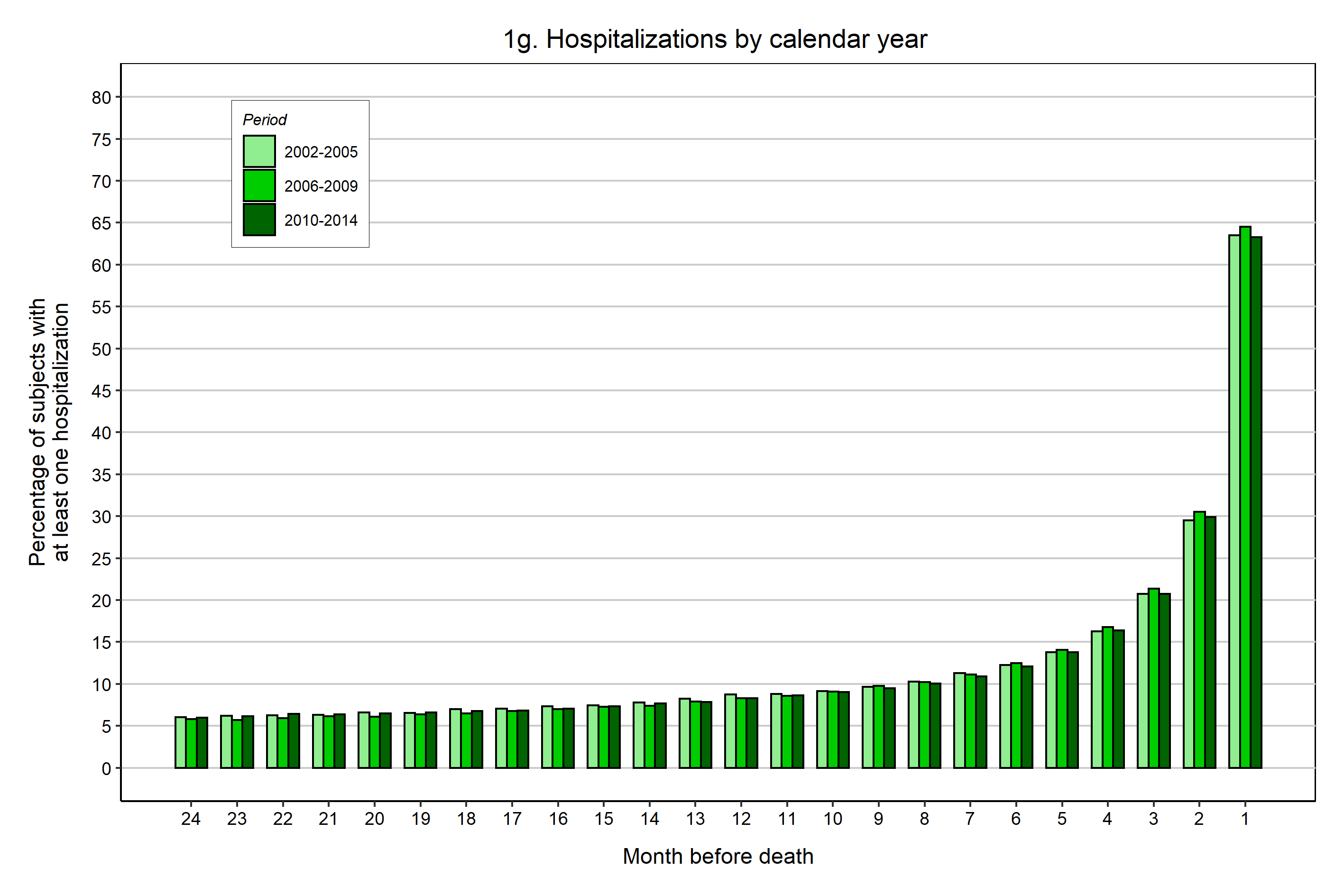 | 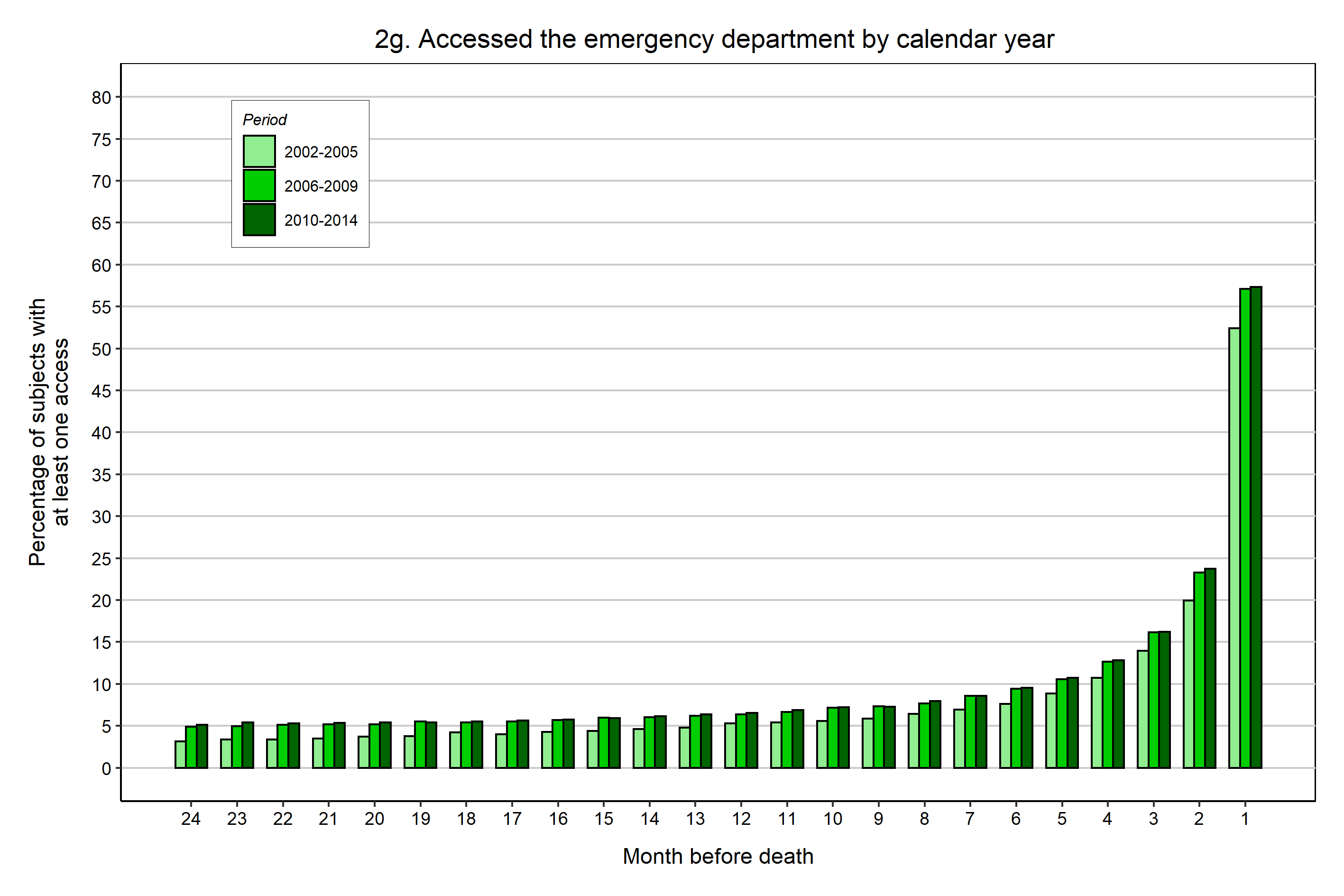 |
| --- | --- |

**Supplemental Figure 2.** Proportion of individuals with an emergency department visit in the 24 months preceding death (excluding nonurgent presentations or minor urgencies).

| 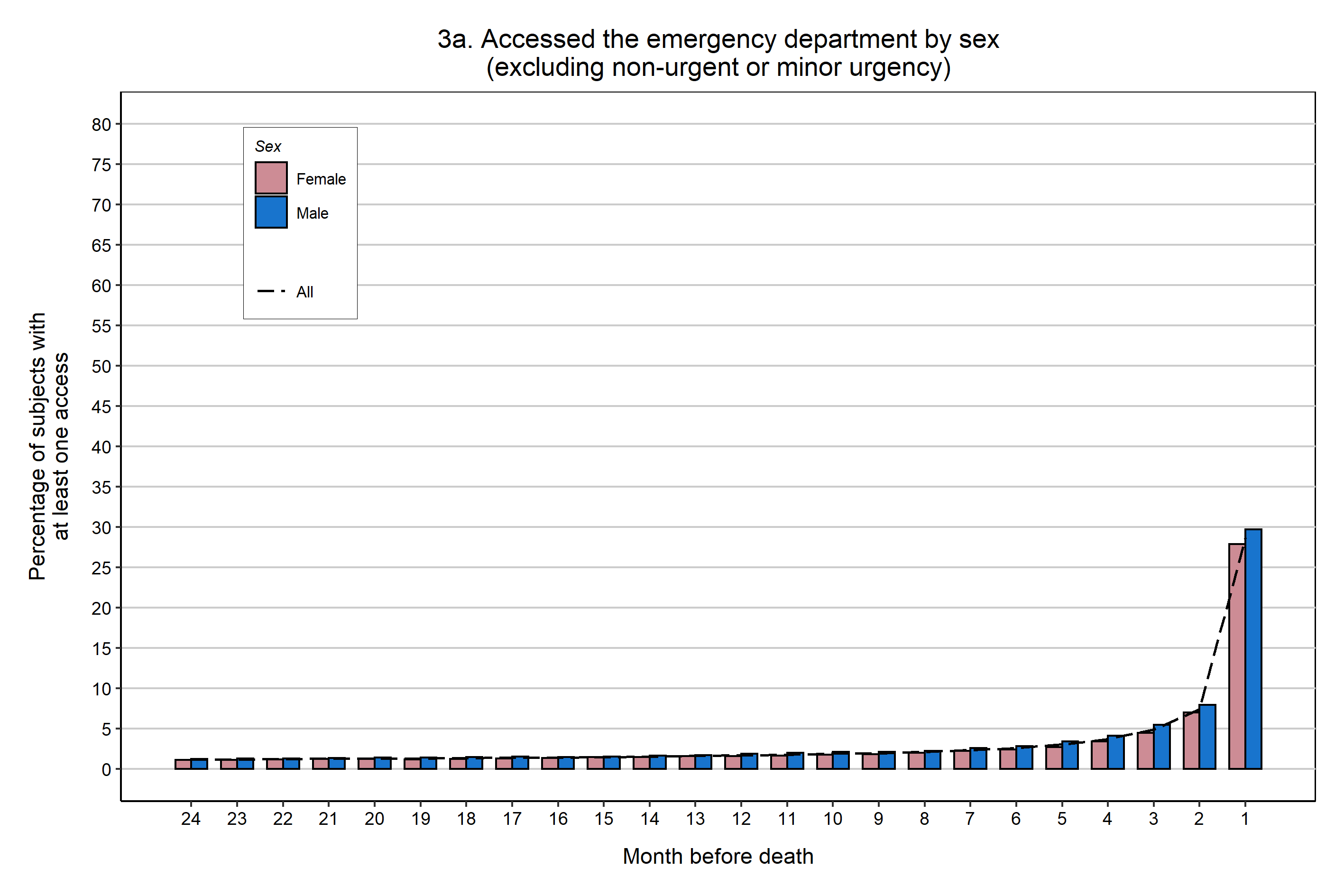 | 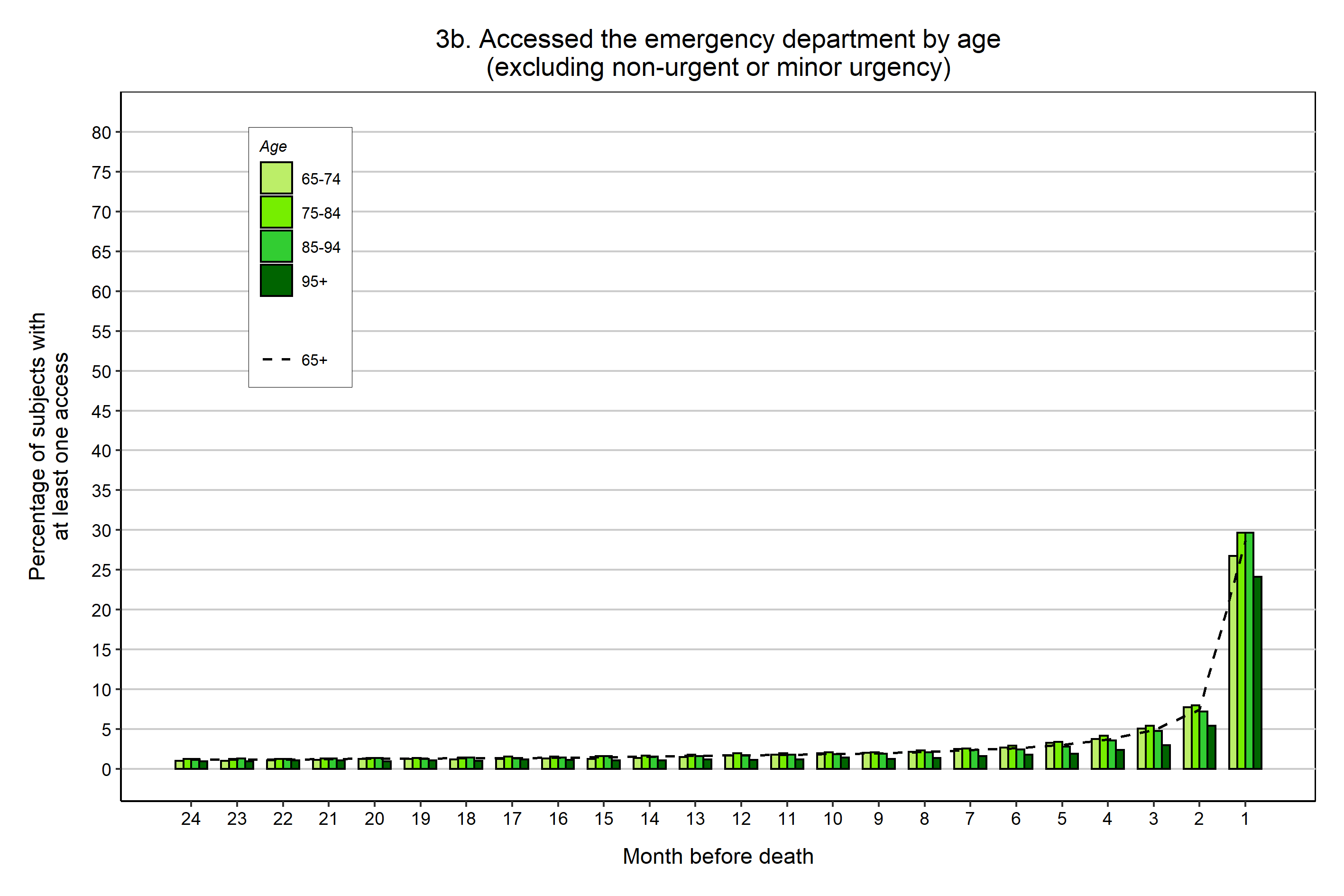 |
| --- | --- |
